# Transarterial Yttrium-90 Radioembolization in Intrahepatic Cholangiocarcinoma Patients: Outcome Assessment Applying a Prognostic Score

**DOI:** 10.3390/cancers14215324

**Published:** 2022-10-29

**Authors:** Imke Schatka, Hans V. Jochens, Julian M. M. Rogasch, Thula C. Walter-Rittel, Uwe Pelzer, Julia Benckert, Josefine Graef, Felix W. Feldhaus, Bernhard Gebauer, Holger Amthauer

**Affiliations:** 1 Department of Nuclear Medicine, Charité–Universitätsmedizin Berlin, Corporate Member of Freie Universität Berlin and Humboldt-Universität zu Berlin, 13353 Berlin, Germany; 2 Berlin Institute of Health (BIH), Charité–Universitätsmedizin Berlin, 10117 Berlin, Germany; 3 Department of Radiology, Charité–Universitätsmedizin Berlin, Corporate Member of Freie Universität Berlin and Humboldt-Universität zu Berlin, 13353 Berlin, Germany; 4 Department of Hematology and Oncology, Charité–Universitätsmedizin Berlin, Corporate Member of Freie Universität Berlin and Humboldt-Universität zu Berlin, 10117 Berlin, Germany; 5 Department of Hepatology and Gastroenterology, Charité–Universitätsmedizin Berlin, Corporate Member of Freie Universität Berlin and Humboldt-Universität zu Berlin, 13353 Berlin, Germany

**Keywords:** Radioembolization, Y-90 microspheres, intrahepatic cholangiocarcinoma, liver metastases, prognostic score, overall survival

## Abstract

**Simple Summary:**

Radioembolization (RE) for intrahepatic cholangiocarcinoma (ICC) is a viable treatment option. As the overall survival (OS) is subject to considerable variance, proper patient selection is of the utmost importance. The current study elucidated overall survival (time from treatment to the patient’s death) in 39 patients after RE in ICC. Out of all the investigated parameters, we identified three pre-therapeutic parameters which were able to predict both OS and patient prognosis by means of a score. More specifically, stratification of the patient cohort by pre-therapeutic GGT, clinical performance status (ECOG) and albumin resulted in significant differences regarding OS (0 risk factors, 15.3 months; 1 risk factor, 7.6 months; ≥2 risk factors, 1.8 months, respectively). Consequently, implementation of this proposed prognostic score may facilitate pre-therapeutic identification of patients who could benefit from RE in ICC.

**Abstract:**

Radioembolization (RE) is a viable therapy option in patients with intrahepatic cholangiocarcinoma (ICC). This study delineates a prognostic score regarding overall survival (OS) after RE using routine pre-therapeutic parameters. A retrospective analysis of 39 patients (median age, 61 [range, 32–82] years; 26 females, 13 males) with ICC and 42 RE procedures was conducted. Cox regression for OS included age, ECOG, hepatic and extrahepatic tumor burden, thrombosis of the portal vein, ascites, laboratory parameters and dose reduction due to hepatopulmonary shunt. Median OS after RE was 8.0 months. Using univariable Cox, ECOG ≥ 1 (hazard ratio [HR], 3.8), AST/ALT quotient (HR, 1.86), high GGT (HR, 1.002), high CA19-9 (HR, 1.00) and dose reduction of 40% (HR, 3.8) predicted shorter OS (each *p* < 0.05). High albumin predicted longer OS (HR, 0.927; *p* = 0.045). Multivariable Cox confirmed GGT ≥ 750 [U/L] (HR, 7.84; *p* < 0.001), ECOG > 1 (HR, 3.76; *p* = 0.021), albumin ≤ 41.1 [g/L] (HR, 3.02; *p* = 0.006) as a three-point pre-therapeutic prognostic score. More specifically, median OS decreased from 15.3 months (0 risk factors) to 7.6 months (1 factor) or 1.8 months (≥2 factors; *p* < 0.001). The proposed score may aid in improved pre-therapeutic patient identification with (un-)favorable OS after RE and facilitate the balance between potential life prolongation and overaggressive patient selection.

## 1. Introduction

Notwithstanding the high frequency of intrahepatic cholangiocarcinoma (ICC) in biliary tract malignancy, it is still accompanied by late diagnosis and dismal outcomes [1]. This is mostly attributable to its commonly asymptomatic progression, being rarely accompanied by jaundice or other signs of biliary obstruction. Consequently, most patients present with advanced stage disease at the time of diagnosis. Even though surgical resection of ICC offers the highest curative potential, many lesions are deemed unresectable at the time of diagnosis. Due to the modest prognosis with median survival rates of less than eight months in unresectable ICC, there is a paucity of alternative treatment modalities [2].

Transarterial radioembolization (RE) with yttrium-90 (Y-90) labeled microspheres has been introduced as a treatment option for treatment-naïve patients with ICC, those with recurrent disease and as a first-line treatment [3,4]. Taking advantage of the radiosensitivity and the unique vascular system of the liver, RE potentially inherits selective high-dose radiation to the target lesion, thus reducing collateral damage to normal liver parenchyma [5,6]. 

Even though current literature suggests that RE is commonly well tolerated, potentially life-threatening side effects have been reported [7]. Radioembolization-induced liver disease (REILD) was seen in up to one third of patients, varying between clinically asymptomatic elevated transaminase levels and hepatic failure [8]. Hence, a meticulous work-up aiding proper patient selection is of particular interest to ensure therapy response. The pre-therapeutic work-up should include pre-therapeutic angiography identifying aberrant vessels or excessive hepatopulmonary shunts and the evaluation of remaining hepatic function [5,9]. Notably, the clinical heterogeneity of ICC has made it difficult to draw definite conclusions on treatment efficacy. Thus, results regarding overall survival (OS) in patients with RE in ICC range from 6.1 to 22 months [10].

Consequently, it is crucial to identify patients who are expected to benefit, while balancing life prolongation and an acceptable quality of life with the hazards of aggressive treatments. Various prognostic scores, using parameters that are consistently associated with (un-)favorable outcomes after RE in different primary liver malignancies or liver metastasis have been described [11,12,13,14,15,16,17,18,19]. Even though several prognostic factors associated with OS after RE in ICC have been proposed—including the Eastern Cooperative Oncology Group (ECOG) performance status, hepatic tumor burden and therapy response according to the RECIST criteria—no adequate prognostic score has been established for RE in patients with ICC yet [20]. Ideally, the latter would only use pre-therapeutic baseline data to ensure that the score can be used to identify suitable patients prior to treatment. 

Therefore, the aim of the present study was to investigate the value of a simple prognostic score in ICC to aid an interdisciplinary assessment of the expected benefits in patients with ICC who are potential candidates for RE.

## 2. Results

### 2.1. Patient Cohort

A total of 39 patients (26 females, 13 males; median age 61 [range, 32–82] years) met the inclusion criteria and were subsequently enrolled in the current study (Table 1). All patients showed evidence of ICC with hepatic metastases. Extrahepatic metastases were present in 20 of the 39 patients (51%). These included lymph node metastases in 19 patients (49%), bone metastases in two patients (5%) and pulmonary metastases in two patients (5%). Regarding the ECOG performance status, 19 patients were categorized as 0, 16 patients as 1 and four patients as 2. Laboratory baseline parameters are listed in Table 1. Pre-therapeutic CA19-9 was only available in 21 of the 39 patients. The interval between [^99m^Tc]Tc-macroaggregated albumin (MAA) scintigraphy and RE had a median of 22 [range, 10–38] days.

Forty-two RE procedures were performed, including 36 (86%) single session treatments with 15 (42%) whole liver/single session treatments and 21 (58%) single lobe/single session treatments. Of these, the planned sequential RE approach in one patient had to be discontinued due to clinical deterioration, and one patient died before undergoing the second sequential RE procedure. Three of the 39 patients (8%) underwent a planned single lobe, sequential RE approach due to impaired liver function (= 6 of 42 RE procedures [14%]). 

Regarding the localization, 17 (44%) whole liver, 13 (33%) right liver lobe and nine (23%) left liver lobe procedures were performed. The median tumor burden relative to the volume of the treated liver lobe (or relative to the total liver volume if it was treated in a single session) was 14.5% (IQR, 4.5–29.9%; range, 1.9–51.6%). 

### 2.2. Overall Survival and Therapy Response

**Figure 1:**
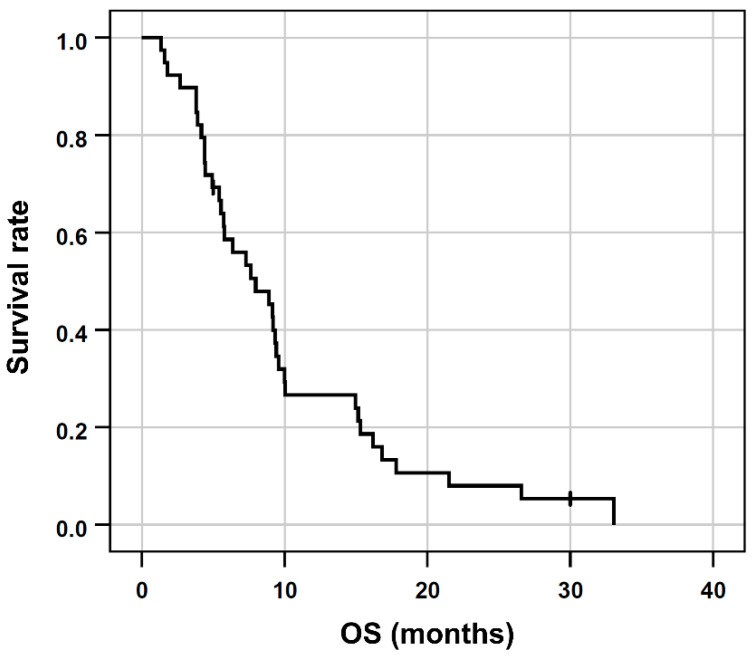
Kaplan-Meier plots regarding OS of the entire patient cohort.

Of the 39 patients, 37 (95%) deceased within the observation period. The median OS after RE was 8 months (95% confidence interval [95% CI], 4.6 to 11.3 months; range, 1.3 to 33.1 months, Figure 1). The follow-up duration in censored patients was 30.1 months.

Hepatic progression-free survival (PFS) was 5.2 months (95% CI, 4.4–6.2; range, 2.9–11.4). At the first follow-up (8.5 weeks after RE (IQR, 7.2 -9.8; range, 5.8–18.2; n = 30)) the response rate according to RECIST 1.1 criteria was classified as partial response in one patient (3%), as stable disease in 28 patients (72%) and as progressive disease in one patient (3%). At the second follow-up (5.2 months (95% CI, 4.4–6.2; range, 2.9–11.4; n = 32)) seven patients (18%) were classified as partial response, 22 (57%) as stable disease and one patient (3%) as progressive disease. Consequently, in the patients with adequate imaging follow-up, the tumor control rate was 97% and the tumor response rate was 23% at both follow-ups. 

### 2.3. Treatment and Toxicity

Even though one patient (3%) showed evidence of microsphere reflux during the RE procedure, post-therapeutic imaging revealed no extrahepatic accumulation. Further therapy-related complications included angina pectoris in one patient (3%) and anaphylaxis due to an allergic reaction to the contrast agent in one patient (3%). After therapy, two patients (6%) developed fever, five (13%) developed abdominal pain and four (10%) developed nausea.

In nearly half of the patients, RE activity was reduced due to excessive hepatopulmonary shunting. A dose reduction of 20% and 40% was required in 14 (36%) and five patients (5%), respectively. A median activity of 1.83 GBq was administered per RE procedure (IQR, 1.4 to 2.0 GBq; range, 0.9 to 2.9 GBq).

### 2.4. Univariable Cox Regression

Concerning metric clinical and laboratory parameters, GGT (HR, 1.002; 95 %-CI, 1.00–1.003; *p* = 0.016), AST/ALT ratio (HR, 1.59; 95 %-CI, 1.08–2.33; *p* = 0.018) and CA19-9 (HR, 1.00; 95 %-CI, 1.00–1.00; *p* = 0.021) were each significantly associated with shorter OS in univariate Cox regression. In addition, higher albumin was associated with significantly longer OS (HR, 0.927; 95 %-CI, 0.86–1.00; *p* = 0.045). Every other parameter, i.e., age, tumor burden, tumor burden of the treated lobe, hepatopulmonary shunting, bilirubin, ASAT, ALAT, INR and ammonia failed to reach statistical significance. 

Concerning ordinal values, dose reduction for RE and higher ECOG status were significantly associated with shorter OS in the univariable cox regression. 

Concerning the nominal values of bilobar or extrahepatic manifestation, the presence of portal vein thrombosis or ascites, single or sequential RE therapy as well as prior therapies showed no significant influence on OS (Table 2).

### 2.5. Multivariable Cox Regression

**Figure 2:**
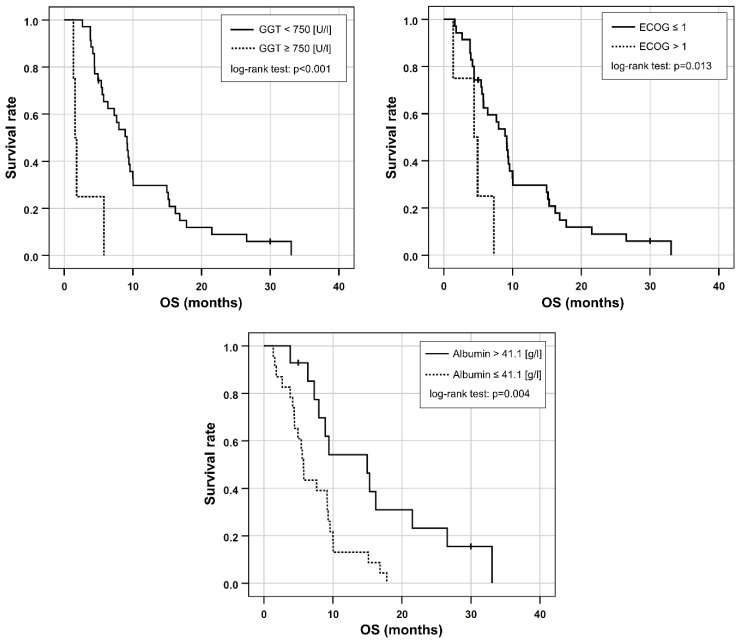
Kaplan-Meier plots for OS in patients separated either by their elevated GGT, ECOG performance status or by albumin reduction at baseline.

Based on the univariable Cox regression, ECOG > 1, AST/ALT ratio ≥ 3.11, the presence of elevated baseline GGT ≥ 750 [U/L], and decreased baseline albumin ≤ 41.1 [g/L] were included in a multivariable Cox regression. As CA 19-9 was only available in 21 patients, it was excluded from further analysis. Dose reduction was not included in the prognostic score because the study aimed to use standard clinical parameters, which would possibly supersede the need for an invasive angiography to perform ^99m^Tc-MAAs scintigraphy as well as RE. Applying stepwise inclusion criteria, ECOG > 1, GGT ≥ 750 [U/L] and albumin ≤ 41.1 [g/L] remained in the final multivariable model (Table 3, Figure 2).

### 2.6. Combined Prognostic Score

**Figure 3:**
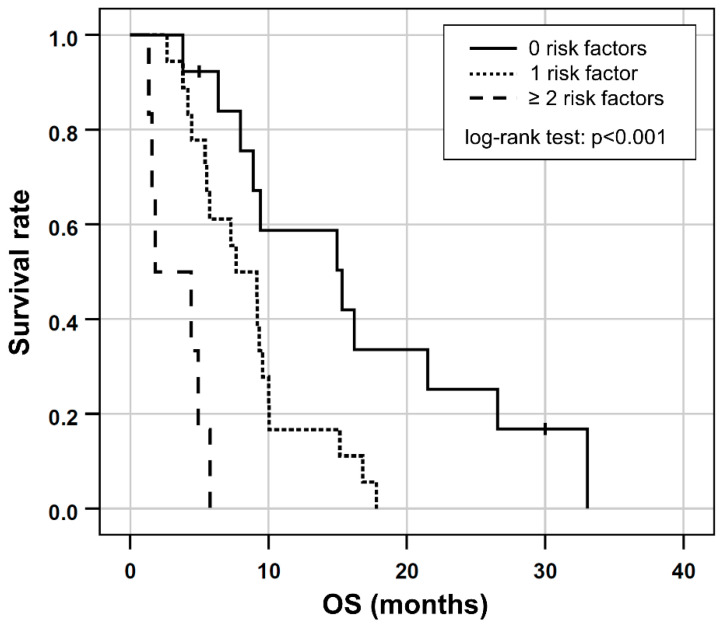
Kaplan-Meier plots for OS in patients separated by the number of risk factors according to the prognostic score based on ECOG > 1 and/or the presence of elevated GGT ≥ 750 [U/L] or low albumin ≤ 41.1 [g/L] at baseline.

Based on the results of the multivariable Cox regression, GGT ≥ 750 [U/L], ECOG > 1 and albumin ≤ 41.1 [g/L] were included in a prognostic clinical score (one point for each risk factor). This combination proved to have the highest prognostic value (Somers’ D: 0.414; Harrell’s C: 0.707) for shorter OS and was consequently chosen as the final score. In the six patients (16%) with two or more risk factors, OS was shorter (median OS, 1.8 months; 95% CI 0.5–2 months) compared to the 18 patients (49%) with one risk factor (median OS, 7.6 months; 95% CI 3.8–11.5 months) or to the 13 patients (35%) with no risk factors (median OS, 15.3 months; 95% CI, 5.5.–25.1 months), respectively, (log-rank test, *p* < 0.001; Figure 3).

## 3. Discussion

Cholangiocarcinoma remains the second most common primary hepatic malignancy [21]. In particular, the incidence of intrahepatic cholangiocarcinoma has increased globally over the last decades. Notwithstanding the potential curative mainstay of surgical resection and liver transplantation, most patients present with advanced stage disease at the time of diagnosis [22]. Proper patient selection remains of particular importance as the inherent clinical heterogeneity of ICC undermines conclusions on eligibility for RE. Even though simple prognostic scores using pre-therapeutic baseline parameters have been proposed for RE in different tumor entities, to the best of our knowledge, no adequate prognostic score has been proposed for RE with Y-90 microspheres in ICC to date. A prognostic score using elevated GGT, ECOG status and reduced albumin baseline levels may facilitate the identification of patients with expected (un-)favorable outcome after RE in routine clinical care.

A growing body of evidence suggests that impaired liver function may negatively impact patient outcomes in ICC. More specifically, an elevated GGT baseline level has been associated with shorter OS in hepatic malignancies [23,24,25]. Moreover, Yin et al. were able to demonstrate that elevated GGT is a predictor of aggressive tumor behavior in ICC [26]. This is in line with the results of the current study, in which GGT ≥ 750 [U/L] proved to be the strongest predictor in the multivariable Cox regression (HR, 7.84) for shorter OS after RE. 

Comparable to our results, ECOG performance has been reported as a predictor of OS after RE in ICC. While Hoffmann et al., Jia et al. and Saxena et al. established cutoff values of ECOG > 0 [20,27,28], Ibrahim and colleagues found the differences between ECOG 0 vs. 1 vs. 2 to be significant [29]. In summary, ECOG seems to reflect a reduced general condition of the patient, which is usually associated with advanced disease, which in turn confers a significantly worse prognosis in ICC. 

Furthermore, low albumin—being the most abundant protein in humans and an integral marker for reduced hepatocellular function—was associated with reduced OS after RE (HR, 3.02). This association has been elucidated in other malignancies [30,31,32]. However, this connection has also been established in the setting of hilar cholangiocarcinoma, revealing advanced stage disease which is deemed potentially life-limiting [33,34].

ICC has a dismal prognosis with an estimated median survival of only a few months. Even though RE with Y-90 microspheres is advocated as a promising treatment option for unresectable ICC, the median survival rates remain less than desirable. A median OS in the current study of 8 months is comparable with previous reports in current literature [27,28,35,36]. Of note, considerably longer OS of up to 22 months has been reported by other authors [20,29,37]. However, these studies included a markedly higher percentage of patients with an ECOG of 0 [37] or applied RE as a first-line therapy [20,38]. Either way, these results show the potential of life prolongation for RE in ICC with precise patient selection. The results of the current study can aid patient selection, as the proposed prognostic score indicated OS of up to a median of 15.3 months if none of the three risk factors were present. In the presence of one risk factor, median OS was only slightly below the overall average (7.6 months). Contrary to this, OS was considerably shorter at only 1.8 months in the presence of all three risk factors. Consequently, patients with two or more risk factors may require especially careful evaluation of the expected benefits. 

Of the three prognostic factors, only the impact of ECOG performance status on OS in ICC has been elucidated in the setting of RE before [20,27,28]. The prognostic role of GGT has only been reported in combination with chemotherapy, hepatectomy or transcatheter arterial embolization in ICC [26] and the relevance of reduced albumin was only described with regard to hilar cholangiocarcinoma [33,34]. Thus, the current results imply additional value in the pre-therapeutic balancing of potential life prolongation with the hazards of invasive treatment.

The analysis of adverse events points out that RE in ICC can be regarded as a relatively safe procedure. Similar to previous reports, the rate of patients who developed clinical toxicity after treatment was low, despite an average therapeutic dose of 1.7 GBq, which falls within average ranges of the reported 1.2 to 2.3 GBq [20,27,28,35,37,38,39,40]. Dose reduction due to excessive hepatopulmonary shunting was necessary in 49% of patients. This raises the question of whether a higher treatment activity could have been administered in our patient cohort. 

Several limitations of the current study need to be acknowledged. Firstly, the retrospective design limits the possibility of a uniform pre- and post-therapeutic treatment protocol. However, we used strict inclusion criteria to compensate for this potential drawback. CA19-9 was only available in a subgroup of 21 patients and was consequently not included in the final prognostic score. It needs to be taken into account that according to univariate analysis it seems to be a promising predictor of outcome. Secondly, this is a single-center study, and therapy algorithms may differ between centers. However, RE was recommended in each patient by interdisciplinary consensus and according to current guidelines [5]. Above all else, the retrospective design may have resulted in under-reporting of adverse events, as it was not possible to retrospectively classify them according to CTCAE criteria. However, patients were closely monitored as inpatients, and the number of patients suffering from clinical toxicity falls into the expected range when compared to similar studies reported in the literature [15,27,28,35,36]. Furthermore, 90Y-PET-CT dosimetry data was not available for this patient cohort [41,42]. Due to the inherent nature of a prognostic score, which is based on pre-therapeutic data, the presented score would not have been changed by dosimetry data. In addition, it needs to be considered that only patients with an activity calculation using mBSA were included, which tends to have a shorter OS compared to the partition model [6]. This choice was made in order to increase data consistency and to reduce the number of confounding variables. Due to a missing control group, prospective, randomized controlled trials are warranted in order to assess the predictive value of the investigated variables beyond their prognostic relevance. 

## 4. Materials and Methods 

### 4.1. Study Design and Eligibility Criteria

This study was approved by the local ethics committee (approval number: EA1/288/16) and carried out in accordance with the Tenets of the Declaration of Helsinki. The institutional database was retrospectively reviewed to identify patients with ICC who underwent RE with Y-90 resin microspheres (vendor: SIRTEX Medical Pty. Ltd., St Leonards, NSW, Australia) between 2009 and 2016. The protocol procedure was in accordance with current guidelines [5]. As published in detail before, interdisciplinary consensus was reached on RE in patients with ICC in the absence of secondary liver malignancies, >18 years of age, as well as with preserved liver function (bilirubin < 2 [mg/dL]) in a salvage situation (either refractory to all accepted therapy regimens at the time of admission or refusal of or non-eligibility for further systemic therapies) [15]. All patients were treated with the best supportive care after RE.

The patient data was reviewed for baseline clinical data including the Eastern Cooperative Oncology Group performance status (ECOG), hepatic and extrahepatic tumor burden, as well as prior liver-directed or systemic treatments. Baseline laboratory markers included the total bilirubin level, serum transaminase levels (alanine transaminase [ALT], aspartate transaminase [AST]), as well as AST/ALT (De Ritis) ratio, albumin, gamma-glutamyltransferase (GGT), alkaline phosphate (AP) and carbohydrate antigen 19-9 (CA19-9). AST/ALT ratio was assessed as proposed by De Ritis et al. [43].

Whole liver volume, or in cases of unilobar RE, the volume of the treated liver lobe, was calculated by pre-therapeutic gadolinium-enhanced magnetic resonance imaging (MRI) using Eclipse (Varian Medical System, Palo Alto, CA, USA). More specifically, manual assessment of the hepatic tumor burden was performed for each liver lobe. CT was performed in the presence of contraindications for MRI, and X-ray or staging CT of the thorax as well as MRI or CT of the abdomen was performed to complete pre-therapeutic imaging. Moreover, this was also used to determine the presence of radiological signs of ascites.

Finally, interdisciplinary consensus was reached for the applied RE activity, a possible reduction by 20% or 40% due to increased hepatopulmonary shunt, unilobar/whole liver RE, and the number of RE sessions.

### 4.2. Radioembolization

RE was performed according to the guidelines of the Radioembolization Brachytherapy Oncology Consortium (REBOC), and as described in detail elsewhere [5,44,45]. The activity of Y-90 microspheres was calculated using the modified body surface area (mBSA) method. The prescribed activity was potentially reduced relative to the BSA method based on lung shunt fraction and tumor involvement [46,47,48]. 

Relative hepatopulmonary shunt volume was assessed by [^99m^Tc]Tc-macroaggregated albumin (MAA) scintigraphy by planar images prior to RE. In the presence of a shunt of 10–15% or 15–20%, the prescribed RE activity was reduced by 20% or 40%, respectively [5]. Whole liver treatment was either performed in a single session or as a sequential protocol, taking the individual risk of post-therapeutic liver failure into account. In any case, the planned activity was administered selectively into the left or right liver lobe, respectively. More specifically, vascular anatomy was assessed angiographically with MAA scintigraphy to delineate shunting to the lung and exclude extrahepatic microsphere deposition. Using the Seldinger technique a 5F vascular sheath was placed preferably in the right common femoral artery. Hereafter, angiography of the celiac trunk and the superior mesentery artery was performed with a 5-F Cobra (Radifocus, Terumo Europe NV) or a 5-F SOS Omni Selective catheter (Soft-Vu, Angiodynamics) and a microcatheter (Cantata 2.5 F or MicroFerret-18 3 F, Cook Medical) was placed into the proper hepatic artery. DSA runs analyzed hepatic artery anatomy, ICC tumor blushes and aberrant arterial blood supply. The latter was coiled—if necessary—to avoid subsequent off-target embolization. After placement of a microcatheter in both the right and the left liver lobe, MAA scintigraphy was performed with 120–200 MBq to delineate hepatopulmonary shunting. Y-90 RE was considered feasible if pulmonary radiation exposure because of hepatopulmonary shunting was below 30 Gy and no relevant extrahepatic deposition was detected.

The radiation dose of Y-90 was calculated based on the modified body surface area (mBSA) and the tumor burden of each lobe as follows:

$$Radiation\;Dose=Body\;surface\;area\;in\;m^{2}-0.2+\frac{\frac{Tumour\;Volume}{Treated\;Liver\;Volume}\;in\;\%}{100}$$

RE was performed analogously in a separate session by the placement of a microcatheter in a lobar liver artery and the application of the calculated radiation dose [45].

### 4.3. Assessment of Toxicity and Survival

Following RE, all patients were monitored as inpatients according to our in-house protocol [15] including daily clinical examination as well as blood works of liver function and blood coagulation. Furthermore, Y-90 SPECT/CT was performed on the first post-therapeutic day to ensure correct hepatic accumulation and to exclude extrahepatic accumulation. According to local protocol, all patients received methylprednisolone, pantoprazole, ursodeoxycholic acid, as well as enoxaprine for 6 to 8 weeks to prevent REILD. 

Patients with symptoms of acute toxicity of RE were treated as inpatients until they showed substantial clinical improvement. Routine follow-up examinations were performed every 3 months, including documentation of the clinical performance status, laboratory works and CT/MRI imaging.

REILD was defined as increased serum total bilirubin (≥3.0 mg/dL) and ascites (grade ≥2 according to the CTCAE) within 12 weeks of RE in the absence of tumor progression or bile duct obstruction.

Overall survival (OS) was calculated as the interval between the date of RE and death from any cause. Therapy response and hepatic progression-free survival (PFS) were assessed using RECIST 1.1 criteria for progressive disease [49]. 

### 4.4. Statistical Analysis

Statistical analysis was performed using SPSS 22 (IBM Corporation, Armonk, NY, USA) as well as R Software (Version 4.1.2, R Foundation for Statistical Computing, Vienna, Austria). Based on the Shapiro-Wilk test, non-parametric data distribution was assumed, and descriptive parameters were expressed as median, interquartile range (IQR) and range. A univariable Cox proportional hazards regression was facilitated to investigate the association of all baseline variables with OS. The liver tumor load was categorized into <25% vs. 25–50% vs. ≥50% tumor load in accordance with previous studies [15,50]. The number of sites of extrahepatic metastases was calculated and included as a metric variable. Variables with *p* ≤ 0.05 in the univariable Cox regression were candidates for inclusion in the multivariable Cox analysis. Care was taken that these variables did not violate the proportional hazards assumption using the goodness-of-fit test (function *cox.zph*) in the survival package of R (The R Project for Statistical Computing, v4.0.2, https://www.r-project.org/) (Accessed on 7 February 2020). Furthermore, the final set of variables in the multivariable Cox regression was determined by stepwise inclusion. Variables that were independent predictors of OS in the multivariable analysis were subsequently included in the prognostic model. The best model was determined based on the highest Harrell’s C or Somers’ D score, respectively. Survival probabilities of the patient cohorts in relation to the (binarized) individual variables and combined score were calculated using the Kaplan-Meier method using the log-rank test and illustrated using Kaplan-Meier curves. Statistical significance was assumed at α = 0.05.

## 5. Conclusions

The current results validate the prognostic value of elevated GGT and ECOG status as well as reduced albumin levels regarding OS after RE in patients with ICC. The proposed three-stage prognostic score is based on routine pre-therapeutic parameters and may aid a targeted patient selection, treatment decision and prognostication. However, further studies are needed to validate these results in a prospective manner.

## Figures and Tables

**Table 1:** Patient characteristics. All percentages are based on the number of patients (n = 39).

Variables	Number (%) or Median (IQR; Range)
RE procedures	42 (100)
Age [years]	61 (IQR, 56–69; range, 32–82)
Female/male sex	26/13 (100)
ECOG performance status	
- 0	19 (49)
- 1	16 (41)
- 2	4 (10)
Baseline laboratory parameters	
- Total bilirubin [mg/dL]	0.5 (IQR, 0.4–0.7; range, 0.1–1.4)
- AST [U/L] - ALT [U/L]	41 (IQR, 35–65; range, 25–394) 25 (IQR, 17–38; range, 12–128)
- AST/ALT ratio	1.8 (IQR, 1.4–2.5; range, 0.73–5.13
- GGT [U/L] - Albumin [g/L] - INR - Ammonia [µmol/L] - CA19-9 [U/mL]	222 (IQR, 91–383; range, 24–1210) 40.6 (IQR, 37.2–42.9; range, 31–47) 1.06 (IQR, 1–1.1; range, 0.92–1.9) 36.2 (IQR, 28.9–52.6; range, 20.2–83.1) 72 (IQR, 8.7–259.8; range, 1.9–36249)
Presence of	
- Extrahepatic tumor manifestation	20 (51)
- Thrombosis of the portal vein	8 (21)
- Ascites - Sequential RE	6 (15) 3 (8)
Baseline hepatic tumor burden	
- unilobar	20 (51)
- bilobar - Liver volume [mL] - Hepatic tumor burden [mL] - Total tumor burden [%] ≤25% 26–50% >50%	19 (49) 1804 (IQR, 1477–2229; range, 947–3560) 316 (IQR, 75–589; range, 22–1838) 14.5 (IQR, 4.5–29.9; range, 1.9–51.6) 27 (69) 10 (256) 2 (5)
Hepatopulmonary shunt [%]	9.8 (IQR, 6.6–12.3; range, 2.6–18.7)
Prior treatment	36 (92)
- Chemotherapy - Hepatic surgery - Local hepatic therapy	29 (74) 21 (54) 8 (21)
- Intraarterial therapy	3 (8)

**Table 2:** Univariable Cox regression. Significant results are printed in bold.

Variable	HR (95% CI)	*p*-Value
Age [years]	1.01 (0.97–1.04)	0.755
ECOG performance status		**0.005**
- 1 vs. 0	1.30 (0.7–2.7)	0.44
- 2 vs. 0	4.27 (1.3–13.8)	**0.015**
- 2 vs. < 2	3.76 (1.2–11.6)	**0.021**
Baseline laboratory parameters		
- Total bilirubin [mg/dL]	0.632 (0.19–2.09)	0.45
- AST [U/l]	1.003 (1.00–1.01)	0.33
- ALT [U/l] - AST/ALT ratio - GGT [U/L] - AP [U/L] - Albumin [g/L] - INR - Ammonia [µmol/L] - CA19-9 [U/ml]	0.999 (0.98–1.02) 1.586 (1.08–2.33) 1.002 (1.00–1.00) 1.002 (1.00–1.00) 0.927 (0.86–1.00) 1.487 (0.21–10.77) 1.005 (0.98–1.04 1.000 (1.00–1.00)	0.88 **0.018** **0.016** 0.77 **0.045** 0.695 0.763 **0.021**
Hepatopulmonary shunt [%]	1.08 (0.99–1.19)	0.09
Dose reduction		
- 20 vs. 0%	1.28 (0.6–2.6)	0.53
- 40 vs. 0%	4.12 (1.2–13.9)	**0.022**
- 40 vs. 0–20%	3.74 (1.2–12.1)	**0.027**
- Total tumor burden [%]	1.01 (0.99–1.03)	0.37
- Tumor burden treated lobe [%]	1.01 (0.99–1.03)	0.28
Presence vs. absence of - Bilobar manifestation	1.12 (0.55–2.08)	0.84
- Extrahepatic tumor manifestation	1.45 (0.74–2.84)	0.29
- Thrombosis of the portal vein	1.08 (0.45–2.39)	0.85
- Ascites - Sequential RE - Chemotherapy - Liver surgery - Local liver therapy - Intraarterial therapy	1.99 (0.80–4.94 0.61 (0.19–1.99) 0.92 (0.49–2.22 0.72 (0.37–1.39) 0.86 (0.37–1.97) 0.39 (0.09–1.68)	0.14 0.41 0.92 0.33 0.71 0.21

HR, hazard ratio; 95% CI, 95% confidence interval.

**Table 3:** Results from multivariable Cox regression after stepwise inclusion of candidate variables. Significant results are printed in bold.

Variable	HR (95% CI)	*p*-Value
GGT ≥ 750 [U/L]	7.96 (2.3–28.1)	**0.001**
ECOG > 1	6.34 (1.8–21.8)	**0.003**
Albumin ≤ 41.1 [g/L]	2.8 (1.2–6.3)	**0.013**

HR, hazard ratio; 95% CI, 95% confidence interval.

## Data Availability

The data presented in this study are available on request from the corresponding author.
